# BEST: Next-Generation Biomedical Entity Search Tool for Knowledge Discovery from Biomedical Literature

**DOI:** 10.1371/journal.pone.0164680

**Published:** 2016-10-19

**Authors:** Sunwon Lee, Donghyeon Kim, Kyubum Lee, Jaehoon Choi, Seongsoon Kim, Minji Jeon, Sangrak Lim, Donghee Choi, Sunkyu Kim, Aik-Choon Tan, Jaewoo Kang

**Affiliations:** 1 Department of Computer Science and Engineering, Korea University, Seoul, Korea; 2 Translational Bioinformatics and Cancer Systems Biology Laboratory, Division of Medical Oncology, University of Colorado Anschutz Medical Campus, Aurora, Colorado, United States of America; Indiana University, UNITED STATES

## Abstract

As the volume of publications rapidly increases, searching for relevant information from the literature becomes more challenging. To complement standard search engines such as PubMed, it is desirable to have an advanced search tool that directly returns relevant biomedical entities such as targets, drugs, and mutations rather than a long list of articles. Some existing tools submit a query to PubMed and process retrieved abstracts to extract information at query time, resulting in a slow response time and limited coverage of only a fraction of the PubMed corpus. Other tools preprocess the PubMed corpus to speed up the response time; however, they are not constantly updated, and thus produce outdated results. Further, most existing tools cannot process sophisticated queries such as searches for mutations that co-occur with query terms in the literature. To address these problems, we introduce BEST, a biomedical entity search tool. BEST returns, as a result, a list of 10 different types of biomedical entities including genes, diseases, drugs, targets, transcription factors, miRNAs, and mutations that are relevant to a user’s query. To the best of our knowledge, BEST is the only system that processes free text queries and returns up-to-date results in real time including mutation information in the results. BEST is freely accessible at http://best.korea.ac.kr.

## Introduction

With biomedical publications increasing in number, knowledge discovery from the literature represents a new challenge for biomedical researchers. Extracting relevant information from a large volume of publications has become an extremely labor-intensive and time-consuming task. Although PubMed serves as a good starting point for researchers, it produces only a list of relevant articles, leaving most of the information-extraction task to the users. For example, PubMed returns 28,924 articles (as of April 14, 2016) for the query “chronic myeloid leukemia.” It is almost impossible for users to sift through all these records to extract relevant information. The problem is exacerbated by the increasing amount of published literature (on average, more than 3,000 articles are added to PubMed every day).

To address this problem, text mining techniques and tools have been developed to assist users.[1] Many biomedical entity search systems have been created to enhance PubMed search. However, the systems have several limitations such as outdated results, slow response time, and limited coverage. First, many existing systems are out of date. To speed up query processing, they preprocess the PubMed corpus to extract information and index the corpus in advance. The PubMed corpus is updated daily and hence new information may not be discovered by existing systems unless they constantly preprocess and index the corpus. Second, many existing systems are slow. Some systems do not preprocess or index the PubMed corpus. Instead, they submit queries to PubMed and process the results returned by PubMed at query time (i.e., each time a user’s query is posted). Consequently, these steps take a long time as the information extraction tasks are done at query time and hence the systems cover only a fraction of the PubMed corpus as the number of articles that can be processed in a given time is limited. Last, many existing systems do not cover all necessary biomedical entities or relations such as mutations, targets, and drugs, to name a few.

More specifically, most previous systems use a conventional search system structure. They extract biomedical entities in indexing time. This scheme speeds up the system at query time. FACTA+ [2,3], DigSee [4], and OncoSearch [5] are index-based entity search systems. Their indices enable them to immediately return query results. However, they can become inconsistent with a source data set. When a source data set (e.g., PubMed) is frequently updated but the systems are not, a search result returned by these systems will not contain up-to-date information or newly discovered knowledge. To resolve this consistency problem due to the systems’ outdated indices, other systems such as Alibaba [6] and PolySearch [7,8] retrieve PubMed abstracts at query time. By this approach, these can use the most recently published articles. Unlike the index-based systems, these systems do not have the consistency problem; however, they process articles after a query is inputted. Thus, these systems take a much longer time to process a user’s query, and cover only a fraction of the PubMed corpus as the number of articles that can be processed in a given time is limited.

To address this challenging problem, we introduce a next-generation biomedical entity search tool (BEST) that directly returns relevant entities rather than a list of documents. BEST returns, as a result, a list of ten different types of biomedical entities including genes, diseases, drugs, chemical compounds, targets, transcription factors, miRNAs, toxins, pathways, and mutations that are relevant to a user’s query. BEST uses a dictionary-based approach to extract biomedical entities from texts, and indexes the entities along with the source texts. The BEST dictionary consists of 12 different databases each covering different subsets of entity types (we will describe it in the Methods section). BEST finds an entity relevant to a query based primarily on the number of co-occurrences between the query terms and the entity in the literature. Besides the co-occurrence, the ranking function of BEST takes into account other various metrics including the authority of journals, the recency of articles, and the term frequency-inverse document frequency (TF-IDF) weighting. To the best of our knowledge, BEST is the only system that processes free text queries and returns up-to-date results including mutation information in real time.

The rest of the paper is organized as follows. In the Results section, we provide an overview of the BEST system, which includes details on querying BEST, identifying entities of various types, the evaluation of entity extraction in BEST, and the performance comparison of BEST and existing systems. In the Discussion section, we explain the differences between BEST and existing systems. In the Methods section, we describe indexing, index update policy, and search and entity scoring methods.

## Results

### Biomedical Entity Search Tool (BEST) System

**Fig 1:**
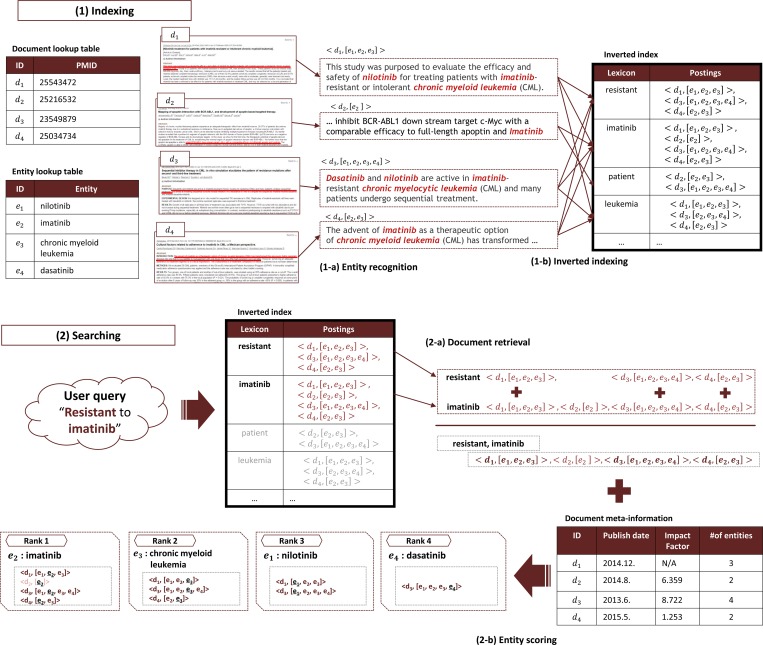
Overview of the BEST System. The BEST system consists of two main parts: Indexing and Searching. (1) “Indexing” represents the indexing subsystem of BEST. For every document, BEST extracts all biomedical entities (1-a) and makes a paired posting (1-b). The basic structure of BEST’s index is similar to that of the inverted index of conventional search engines. However, BEST uses a different indexing unit, paired posting, which is a pair of a document ID and a list of entities that appear in the document. (2) “Searching” represents the search subsystem of BEST. All retrieved paired postings are aggregated to rank the entities (2-a). Ranking scores are computed using four subcomponents described in “Searching and Scoring” in the Methods section (2-b).

We developed BEST on Apache Solr v.4.9, a Lucene-based search platform. We logically redefined Solr’s indexing structure and ranking system to score entities. Fig 1 is an overview of the BEST system. BEST can be divided into two parts: Indexing (Fig 1(1)) and Searching (Fig 1(2)). BEST indexes not only PubMed abstracts, but also biomedical entities described in the abstracts. The pair of abstract and entity list is indexed as a posting in the inverted index. This indexing scheme was originally introduced in our prototype system BOSS [9]. BEST extends the original indexing system with the auto-update feature to synchronize with PubMed. BEST improves the accuracy of search with the enhanced coverage of indexed entity types and its new ranking algorithm.

BEST indexes the entire MEDLINE/PubMed corpus that consists of more than 25 million abstracts and continues to grow daily as more articles are added to the corpus. To keep its database up to date, BEST is programmed to visit the PubMed FTP site once every day and update its index by incorporating articles that were newly added to the PubMed corpus. There exist other archives with full-text articles (e.g., 3.9 million articles in PubMed Central). However, the current version of BEST is designed to search only the 25 million abstracts (just like PubMed) for the following two reasons: 1) we want the search coverage of BEST to be the same as that of PubMed; and 2) indexing all full-text articles and updating them daily requires a considerable amount of computing resources. However, we are aware of the importance of using full-text articles. In our future work, we will investigate how to extend BEST so that it can search full-text articles while keeping the system cost low.

Due to the paired posting list of BEST’s index, the system can immediately return biomedical entities from a query. First, BEST obtains all the postings from the inverted index that matched the query terms. Second, BEST groups the retrieved postings by entities contained in the postings. Last, BEST scores each entity by aggregating the scores of the postings in the corresponding entity group. The BEST system architecture and the scoring scheme is further detailed in the Method section. BEST is freely available at http://best.korea.ac.kr.

### Querying BEST

Similar to PubMed, BEST answers any ad-hoc keyword query. Even though a query does not include any biomedical entities, BEST returns a list of entities related to the query. BEST also supports Boolean queries. Here, we illustrate some query examples, and the results returned by the BEST system.

#### Identifying genes and drugs using BEST

**Fig 2:**
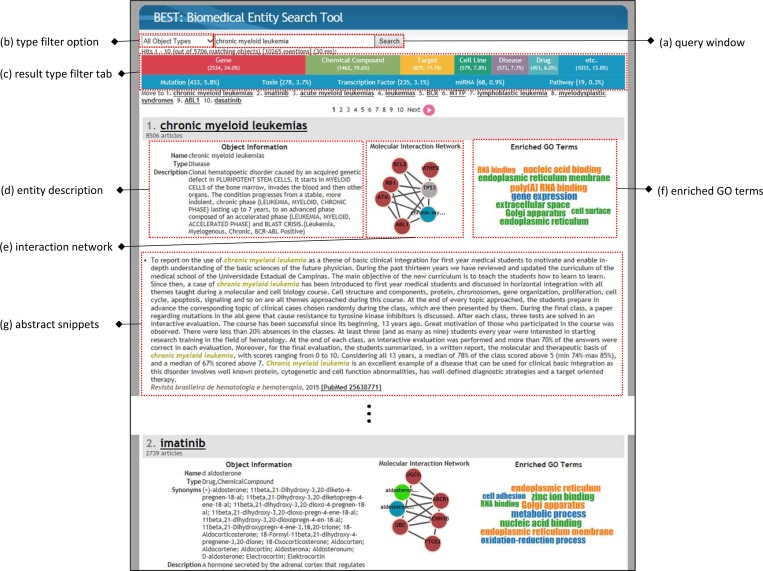
BEST Interface. Users can pose queries in the query window (a) and select result entity types either by using the drop down box (b) or by clicking on the entity-type filter tab (c). BEST returns a list of entities that are relevant to a user’s query. For each entity in the list, BEST shows a description (d), an interaction network (e), enriched GO terms (f) of the entity, and top 3 abstracts (g) in which the query terms and the entity co-occur.

To illustrate the functionality of BEST, we use chronic myeloid leukemia (CML) as a running example. CML is a type of leukemia that is caused by the BCR-ABL oncogene which is due to chromosomal rearrangement. To query the mutations and drugs related to CML, a user can query “chronic myeloid leukemia” in BEST. BEST returns a list of entities including the disease itself and imatinib as the first and second results, respectively (Fig 2). Imatinib (Gleevec) is the first approved targeted therapy for CML. Additional information provided with each entity includes a description of the entity (Fig 2(D)), a molecular interaction network centered around the entity (Fig 2(E)), computed enriched GO terms from the abstracts that match the entity (Fig 2(F)), and three abstract snippets with highlighted query terms and the entity (Fig 2(G)). Users can obtain more detailed information about a particular entity by clicking on the entity’s name, which will lead to an entity page where users can access all the abstracts in which the query terms and the entity co-occur.

#### Identifying mutations that confer resistance to drugs

In CML, acquired mutations in ABL1 can lead to imatinib resistance. Suppose a user would like to know about the mutations that confer resistance to imatinib in CML. The user can select “Mutations” from the dropdown box as the desired result type and search “imatinib resistance ABL1” (Fig 2(A) and 2(B)). Table 1 lists the top 10 results from the query. Among the top 10 mutations returned by BEST, mutations T315I, Y253H, and V289F were found to be the binding sites of imatinib [10,11]. These mutations are acquired gatekeeper mutations that result in drug resistance. Notably, all three mutations were previously demonstrated to confer resistance to imatinib, and found in CML patients resistant to imatinib.[11,12] However, PubMed will return 190 articles for the same query (as of April 14, 2016), and the user still has to go through these articles to extract the ABL1 mutations that confer resistance to imatinib in CML. This result demonstrates that the user can use BEST to gather and summarize information scattered across multiple articles.

**Table 1:** Search result of query "imatinib resistance ABL1" with type filter "mutations."

Rank	BEST result	BEST score	ABL1 mutation	Known Imatinib binding site in ABL1 [10,11]	Acquired mutations found in CML patient resistant to imatinib
1	**T315I**	24.520	Yes	Yes	Yes
**2**	**Y253H**	7.744	Yes	Yes	Yes
**3**	**E255K**	5.602	Yes		Yes
**4**	**E355G**	2.871	Yes		Yes
**5**	**G250E**	2.757	Yes		Yes
**6**	**G398R**	2.048	Yes		Yes
**7**	**M351T**	1.430	Yes		Yes
**8**	**Q252H**	1.349	Yes		Yes
**9**	**E255V**	1.328	Yes		Yes
**10**	**V289F**	1.218	Yes	Yes	Yes

#### Identifying alternative drugs that overcome acquired resistance

Users can also apply an entity type filter by clicking on one of the entity type tabs (Fig 2(C)). If a user would like to know about alternative drugs for patients who are resistant to imatinib, the user can simply click on the “Drug” tab in the result page for drugs such as nilotinib, dasatinib, bosutinib, and ponatinib. These drugs have been found to be effective alternatives.[13] For example, in one of the abstracts returned from the query “nilotinib,” a user can find an alternative therapeutic choice for CML patients resistant to imatinib as follows.

“In recent years, several second-generation inhibitors—such as dasatinib and nilotinib—have become available, these promise to overcome some of the mutations associated with acquired resistance to imatinib.” [PubMed ID: 25216683]

#### Identifying related genes in a pathway

**Fig 3:**
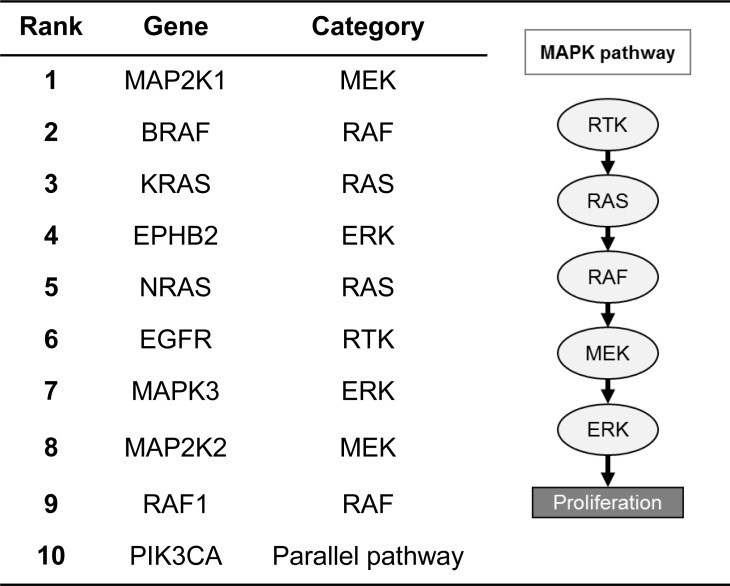
BEST’s result of "MAP2K1" with type filter "genes."

By querying “MAP2K1,” a gene involved in the MAPK pathway, and clicking on the “Genes” tab, BEST will return all related genes involved in the MAPK pathway. Fig 3 shows the top 10 gene results from the query. As expected, the top nine genes are the known members of the classical signaling cascades (RTK, RAS, RAF, MEK, and ERK) of the MAPK pathway. Interestingly, the tenth gene returned from this query is PIK3CA, a gene not directly involved in the MAPK pathway but is a known core member of the parallel PI3K/AKT/MTOR signaling pathway. As presented in this result, BEST returns a series of genes that are relevant to the query.

These simple steps provided by BEST allow the user to navigate the retrieved information from the initial query and discover new knowledge. We believe these features distinguish BEST from other existing biomedical text mining systems. Users can retrieve articles from PubMed but they have to read the returned abstracts to discover information. For a more detailed explanation about the BEST query interface and additional use cases, please refer to the BEST manual at http://best.korea.ac.kr/help/BEST_Guide.pdf

### Evaluation of biomedical entity extraction in BEST

Next, we evaluate the precision and recall of BEST’s entity extraction module using the Biomedical entity Relation ONcology COrpus (BRONCO).[14] BRONCO-A is a corpus that consists of 108 oncology-related PubMed abstracts containing annotations of gene, disease, drug, and cell line information. BEST uses its own dictionary-based named-entity extraction module which is available at http://infos.korea.ac.kr/bioentityextractor/ (see the Methods section for details). We compared BEST’s entity extraction module with PubTator[15] which is a state-of-the-art machine-learning-based biomedical entity extraction tool. We evaluated the performance of BEST and PubTator in extracting genes, chemical compounds (drugs), and diseases from BRONCO-A. BEST’s entity extraction module achieves a precision of 92.80%, a recall of 69.06%, and an F1-score of 79.18%. In comparison, PubTator’s precision, recall, and F1-score are 87.26%, 74.59%, and 80.43%, respectively. This comparison demonstrates that BEST’s biomedical entity extraction module achieves results comparable to those of PubTator.

### Comparing BEST with Existing Systems

To evaluate the performance of BEST, we compared it with FACTA+[3] and PolySearch2,[8] the two most recent biomedical entity search systems. We chose FACTA+ and PolySearch2 because they support ad-hoc query search and multiple types of biomedical entities. Similar to BEST, the two systems use PubMed as their source data. We qualitatively compared BEST with FACTA+ and PolySearch2 using various queries.

FACTA+ and BEST construct their indexes using PubMed abstracts. BEST automatically updates its index daily; however, FACTA+ has not updated its index since the launch of the service, producing outdated results. PolySearch2 analyzes retrieved abstracts at query time, while the other systems do not conduct query-time analysis. Due to the query time analysis, PolySearch2 requires a much longer query processing time than the others. For the test with the query “chronic myeloid leukemia,” PolySearch2 took more than 30 seconds to return the result (cold-cache query). To improve query response time, PolySearch2 utilizes cache. Once a user inputs a query, the system caches the result for later use. However, due to the constant addition of new articles to PubMed, the cached results must be frequently invalidated, limiting the benefits of using cache. Table 2 lists the query response time of the three systems for the query “chronic myeloid leukemia.” As Table 2 shows, FACTA+ is the quickest system in returning the results, and is followed by BEST. PolySearch2 has the longest query response time.

**Table 2:** Top 10 drugs returned for query "chronic myeloid leukemia."

		BEST	PolySearch2	FACTA+	FDA approved drugs for CML*
Query response time		0.024 s	30 s	0.01 s	
**Rank**	1	**Imatinib**	**Imatinib**	**Imatinib**	**Bosutinib**
	2	**Dasatinib**	**Busulfan**	**Gleevec**	**Busulfan**
	3	**Nilotinib**	**Dasatinib**	**Dasatinib**	**Cyclophosphamide**
	4	Interferon alpha	**Nilotinib**	*Progesterone*	**Cytarabine**
	5	**Hydroxyurea**	**Hydroxyurea**	**Bosutinib**	**Dasatinib**
	6	**Busulfan**	*Cocaine*	Bortezomib	**Hydroxyurea**
	7	**Cyclophosphamide**		Valproic Acid	**Imatinib**
	8	**Cytarabine**		Glutathione	**Nilotinib**
	9	**Bosutinib**		*lysine*	**Omacetaxine mepesuccinate**
	10	Fludarabine		Flavopiridol	**Ponatinib**
Number of retrieved FDA approved drugs for CML		**8**	**5**	**3 (Gleevec is brand name for Imatinib)**	

*Note: Source: http://www.cancer.gov/about-cancer/treatment/drugs/leukemia#7

To evaluate the accuracy of the systems, we focus on identifying relevant drugs for “chronic myeloid leukemia” in the results. The top ten drugs retrieved by the three systems were tabulated in Table 2. For evaluating the results of each system, we obtained the list of ten FDA approved drugs for CML from the National Cancer Institute’s drug information page [16]. Out of the top ten drugs returned by BEST, eight are FDA approved for CML. In contrast, FACTA+ returned only three FDA approved drugs from its top ten list (note: Gleevec is the brand name for imatinib). PolySearch2 returned only six drugs out of which five are FDA approved for CML. While evaluating the BEST results, we found that hydroxyurea is also an FDA approved drug used in treating CML in the chronic phase, and has demonstrated better efficacy than busulfan.[17] Another drug retrieved by BEST is fludarabine, which is an FDA approved drug for chronic lymphocytic leukemia. This demonstrates that the results returned by BEST are all relevant drugs for CML. However, not all of the drugs that were returned by FACTA+ and PolySearch2 are used for chronic myeloid leukemia. For example, progesterone and lysine were in the FACTA+ results and cocaine was in the PolySearch2 results.

For a concrete evaluation, we tested three systems using more queries. Each query is expected to return a list of drugs that are related to the query. For a disease query, we expect a list of treatments of the disease, and for a drug query, we expect the drugs that belong to the same category. For each query, we checked how many correct biomedical entities are returned. Table 3 shows the summarized results. Moreover, the detailed query results are presented in Table A, B, and C in S1 File. Briefly, BEST achieves the best performance. PolySearch2 returns relatively accurate results, but it takes more than 30 seconds for each query. FACTA+ immediately returns results but the results were not as relevant to the query as those returned by the other systems.

**Table 3:** Accuracy and response time comparison of Best, PolySearch2, and FACTA+.

	BEST		PolySearch2		FACTA+	
Query	Precision@10	Response time	Precision@10	Response time	Precision@10	Response time
“chronic myeloid leukemia”	**0.8**	0.024s	0.5	30s	0.3	0.01s
“lung cancer”	**0.9**	0.116s	0.9	30s	0.4	0.09s
“melanoma”	**0.5**	0.058s	0.1	28s	0.1	0.07s
“tyrosine kinase inhibitor”	**0.9**	0.067s	0.8	45s	0.2	0.03s
**Average**	**0.76**	0.066s	0.58	33.25s	0.23	0.05s

We can conclude that BEST returns the most relevant results for the query, followed by PolySearch2 and FACTA+.

### Evaluating the recency factor in BEST

**Fig 4:**
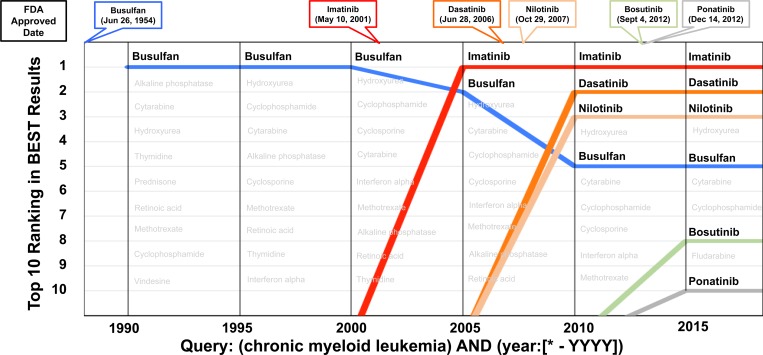
Recency evaluation of BEST using "(chronic myeloid leukemia) AND (year:[*—YYYY])" with result type filter “drug.”

To demonstrate the utility of the recency factor in BEST, we query the drugs that were retrieved for “chronic myeloid leukemia” from 1990 to 2015 with a 5-year interval (i.e., from all articles up to 2000, all articles up to 2005, etc.). As illustrated in Fig 4, we found that the ranking of the drugs changed over this period of time. For example, busulfan, a chemotherapy agent approved in 1954 by the FDA, was ranked as the top drug result for the query “chronic myeloid leukemia” from 1990–2000. However, since the introduction of imatinib in 2001, we have entered into the targeted therapy era for cancer treatment. In 2005, imatinib was ranked as the number one drug for chronic myeloid leukemia whereas busulfan was ranked number two. Dasatinib (2006) and nilotinib (2007), both of which are second-generation targeted therapies for CML, ranked higher than busulfan in the 2010 results. From the 2015 results retrieved by BEST, we observed an increase of bosutinib and ponatinib drugs both of which are new-targeted therapies that were approved for CML in 2012. This demonstrates that BEST uses the recency factor for ranking results, reflects up-to-date knowledge, and uncovers research trends in the biomedical literature.

We also evaluated how the search result changes if we remove or boost the weight of the recency component in our scoring scheme. We control the impact of the recency factor by the recency component in our scoring formula (refer to the Methods section for details about the BEST scoring function). For example, if the recency component is raised to the fourth power, then its value will be weighted four times more than that of the other scoring components. On the other hand, if the power is zero, the recency factor is not used in scoring. The default value (as used in Fig 4) is one. As presented in Table 4, when we give more weight to the recency factor, drugs that have been approved more recently, e.g., ponatinib or bosutinib, are ranked higher in the list. Busulfan, the old-fashioned treatment of CML, is ranked lower. This result supports our argument that the recency factor is useful to a user who wants to get up-to-date knowledge. We implemented the “tunable scoring function” which can be accessed in the “Advanced search” option. By this option, users can customize the weights of scoring components according to their search purposes.

**Table 4:** Search results of drugs when more weight is given to the recency factor.

	Power of recency			
Rank	0	1.0	2.0	4.0
**1**	Imatinib	Imatinib	Imatinib	Imatinib
**2**	Dasatinib	Dasatinib	Dasatinib	Dasatinib
**3**	Nilotinib	Nilotinib	Nilotinib	Nilotinib
**4**	Interferon α	Interferon α	Bosutinib	Bosutinib
**5**	Hydroxyurea	Hydroxyurea	Hydroxyurea	Ponatinib
**6**	Busulfan	Busulfan	Ponatinib	Hydroxyurea
**7**	Cytarabine	Cyclophosphamide	Busulfan	Cyclophosphamide
**8**	Cyclophosphamide	Cytarabine	Fludarabine	Fludarabine
**9**	Fludarabine	Bosutinib	Cyclophosphamide	Busulfan
**10**	Methotrexate	Fludarabine	Interferon α	Homoharringtonine

## Discussion

We developed BEST, a next-generation biomedical entity search tool for knowledge discovery in PubMed. The BEST system utilizes sophisticated text-mining approaches to extract biomedical entities from PubMed abstracts. Using a novel indexing and scoring scheme, BEST indexes 10 different types of biomedical entities (e.g., genes, diseases, drugs, targets, transcription factors, miRNAs, and mutations) from the entire PubMed corpus. BEST processes users’ free text queries and returns up-to-date results in real time. As we have demonstrated in several examples, BEST is capable of returning relevant results for queries. Results were evaluated against known knowledge, and found to be accurate and relevant. Furthermore, BEST outperform the other systems in returning relevant results for queries.

Most of the systems developed previously focused on a limited number of entity types or knowledge context. Consistency with PubMed, query processing time, and coverage of knowledge as well as the generality of information are important features for biomedical entity search systems. However, existing systems lack at least one of these features. BEST is different from the existing tools for the following reasons: (i) it is fast and constantly up to date; (ii) returns rich information including genes, mutations, diseases, drugs, and targets; and (iii) returns the relations between these entities. The main features of the existing systems are presented in Table D in S1 File. No existing system has both the consistency and real-time response features. Unlike the existing systems, BEST automatically updates its index and preprocesses rich information from the PubMed corpus daily, and thus has both the consistency and real-time response capability. Moreover, retrieving genomic variants from the literature is one of the unique features of BEST that is not supported by the other systems.

When we extract named entities from massive text data, we consider precision more important than recall because low recall can be resolved by integrating results from a very large amount of source text. However, if the results are not precise, the integration of these results will not be useful for inference tasks. For this reason, BEST employs a dictionary-based approach for extracting entities. We want BEST to achieve high precision and reasonable recall in extracting biomedical entities from text. As demonstrated in the Results section, BEST’s entity extraction module performance on the BRONCO-A corpus was comparable to that of PubTator. Like PubTator, BEST’s entity extraction module can extract genes/proteins, chemical compounds (drugs), and diseases from text. We also implemented tmVar[18] for extracting genetic variation information from the text, similar to PubTator’s mutation module. In addition, BEST extracts cell lines, kinases, toxins, miRNAs, and pathways, whereas PubTator does not.

In summary, BEST assists users in searching and linking biomedical entities in the literature. BEST is programmed to update its database daily to remain consistent with PubMed. All PubMed abstracts are indexed with a paired posting of documents and entity list so that BEST can immediately retrieve relevant biomedical entities. BEST scores all biomedical entities and returns them to a user as a ranked list using a novel scoring method. When scoring an entity, BEST considers the co-occurrence frequency between query terms and the entity, recency of an article, the reputation of a journal, the number of entities in a document, and the conventional information retrieval score. BEST is a unique system because it contains the latest published information and it can immediately return results.

## Methods

### BEST System Architecture

BEST is built on Apache Solr v.4.9, a Lucene-based search platform, where the Solr’s indexing structure and ranking system were logically redefined to score entities. The BEST system can be divided into indexing and searching subsystems.

### Indexing Subsystem

BEST indexing subsystem performs entity extraction, entity indexing, and meta-information indexing. The detail of each step is given below. The index size of BEST is 34.22 GB, which includes 11,882,670 abstracts. The 282,936 entities are extracted from the abstracts. More statistics are provided in Table E in S1 File.

#### Dictionary based named entity recognition

We used a dictionary-based approach to extract entities from text (Fig 1(1-a)). To construct our entity dictionary, we integrated biomedical entity names from 12 different sources as listed in Table 5. We also used cross-reference IDs from each database to deal with identical entities in different entity databases. If there was a pair of entities that shared the same reference ID, they were considered as synonyms and merged into a synonym set. To resolve potential type assignment conflicts, we categorized biomedical entities into the following four groups: gene_group, chem_group, disease_group, and pathway_group. In the gene_group, genes/proteins, targets, transcription factors, and miRNAs are included. Chemical compounds, drugs, and toxins are included in the chem_group. Diseases and pathways are included in the disease_group and the pathway_group, respectively. One entity can be multiple types within a group but not across the groups. For example, TP53 can be a “gene/protein” entity type and a “transcription factor” entity type, simultaneously. To avoid missense type assignments, BEST prioritizes the entity groups. The gene_group has the highest priority followed by the chem_group, disease_group, and pathway_group. If an entity from a source erroneously has multiple types from different entity groups, BEST will assign the more prioritized type to the entity. After integrating entity names from the 12 sources, human reviewers manually checked the correctness of the contents of the dictionary. The entity extraction module was developed independently, and is available at http://infos.korea.ac.kr/bioentityextractor/.

**Table 5:** Source databases for BEST dictionary.

Entity Type	Source Databases (URL)	Entity Group
Gene/Protein	NCBI Entrez Gene (http://www.ncbi.nlm.hig.gov/gene)	Gene
Target	DrugBank (http://www.drugbank.ca/)	Gene
	T3DB (http://www.t3db.ca/)	
Transcription Factor	Animal TFDB (http://www.bioguo.org/AnimalTFDB/)	Gene
	Therapeutic Target Database (http://bidd.nus.edu.sg/group/cjttd/)	
miRNA	miRBase (http://www.mirbase.org/)	Gene
Chemical Compound	PubChem (http://pubchem.ncbi.nlm.nih.gov/)	Chem
Drug	DrugBank (http://www.drugbank.ca/)	Chem
	US FDA Approved drugs (http://www.fda.gov/)	
Toxin	T3DB (http://www.t3db.ca/)	Chem
Disease	MeSH (http://www.ncbi.nlm.nih.gov/mesh)	Disease
Pathway	KEGG Pathway (http://www.genome.jp/kegg/)	Pathway

#### Document-entity list pair indexing

After recognizing entities from PubMed abstracts, BEST indexes the entities and abstracts (Fig 1(1-b)). Similar to other document search engines, BEST indexes all the words in the abstracts. However, unlike conventional information retrieval systems, BEST indexes the link between the abstracts and the biomedical entities that appeared in the abstracts. BEST pairs abstracts with lists of entities that appear in the abstracts, and each pair is considered as one posting. For example, from a document containing the sentence, “Consistent use of imatinib is critical for treatment success in chronic myeloid leukemia.…,” (PMID: 24524212), BEST makes the posting <PMID: 24524212, [imatinib, chronic myeloid leukemia]> for indexing. In this posting, two entities (imatinib and chronic myeloid leukemia) are included in the list. All the terms in the articles (such as consistent, use, imatinib, critical, treatment, success, and so on) will be the keys of the index where each key indicates this posting.

#### Entity meta-information and index update policy

As a query result, BEST returns not only a list of entities but also the information of each entity such as the molecular interaction network of each entity, enriched Gene Ontology (GO) terms, and frequent n-gram phrases that co-occurred with an entity (Fig 2 and Fig A in S1 File). This information is called the meta-information of an entity. The molecular interaction network of each entity, which visualizes the relationships to other entities, is shown as a graph and is extracted using Biomedical Entity-Relationship eXplorer (BEReX).[19] We calculate the enriched GO terms using the graph and a method proposed by Eden et. al.[20] In this method, a set of genes is required to compute enriched GO terms for each entity; we used the genes adjacent to the entity in the BEReX graph. This information is indexed in indexing time. However, frequently co-occurring n-gram phrases are computed at query time since they are dependent on documents retrieved for each query (Fig A-(a) in S1 File). Last, the related keywords are extracted from the “related substances” field in the PubMed records (Fig A-(b) in S1 File). The keywords are sorted based on the frequency in retrieved documents. This information is computed at indexing time.

BEST automatically downloads abstracts newly indexed in the PubMed system and updates its index every day. As a result, BEST can remain consistent with the PubMed system.

### Searching and Scoring Subsystem

#### Document retrieval

When users input a query, BEST searches its index for documents containing the query terms (Fig 1(2-a)). This process is similar to that of general search engines. After the query is input, the system looks through the inverted index to retrieve postings that match the query terms. BEST supports conventional search operations such as Boolean operators, proximity search, and term boosting. Fig 1(2) depicts the searching process for the query “resistant to imatinib.” First, BEST parses the query string. In this example, BEST removes the stop word “to” from the query string. In the next step, BEST tokenizes each query term and retrieves all the postings that match them. In this example, d1, d2, and d3 are retrieved using the term “resistant” and d1, d2, d3, and d4 are retrieved using the term “imatinib.” For documents d1 to d4 and the postings, refer to Fig 1(1).

#### Entity scoring

BEST calculates the scores of all matching entities for a query, as illustrated in Fig 1(2-b). All entities retrieved by the query are ranked according to their integrated entity scores. The score of an entity is computed as the sum of the scores of all documents that contain the entity and the query terms. The entity score *S*_*e*_(*q*) is formally defined as

$$S_{e}(q)=\sum_{d\in D_{e}(q)}S_{d}(q)$$

where *S*_*d*_(*q*) is the score of document *d* with respect to query *q* and *D*_*e*_(*q*) is the set of documents containing both query *q* and entity *e*. The document score *S*_*d*_(*q*) is a product of four components and is defined as

$$S_{d}(q)=T_{d}(q)\times N_{d}\times Q_{d}\times R_{d}$$

where *T*_*d*_(*q*) is the term match score of document *d* with respect to query *q*; *N*_*d*_ is the entity number score; *Q*_*d*_ is the source reputation score; and *R*_*d*_ is the recency score of document *d*. The details of the four score components are given below.

#### 1) Term match score (*T*_*d*_(*q*))

The term match scoring scheme of BEST is similar to that of general document search engines. To compute the term match score, term frequency (TF) of a query word in a document, inverse document frequency (IDF) of a query word in a corpus, and the overlap between the query words and the document (coord) are used. The term match score *T*_*d*_(*q*) is defined as

$$T_{d}(q)=coord(q,d)\times tfidf(q,d)$$

where *coord*(*q*,*d*) represents the overlap between query *q* and document *d*, which ranges from 1/|*q*| to 1, and *tfidf*(*q*,*d*) is the sum of TF-IDF weights of the terms in query *q* with respect to document *d*.

#### 2) Entity number score (*N*_*d*_)

We give penalties to articles that contain many entities. If an abstract contains only one entity, most of the sentences in the abstract may be related to the entity. However, if an abstract contains many entities, the contribution of the abstract toward the entity score must be proportionally reduced. The entity number score ranges from 1/10 (lower bounded) to 1 and is defined as

$$N_{d}=\frac{1}{min(|E_{d}|,10)}$$

where *E*_*d*_ is the number of entities in document *d*.

#### 3) Reputation score (*Q*_*d*_)

BEST considers the reputation of the source journal of each article. According to [21], a journal’s reputation can be considered to help users prioritize search results. The impact factor (IF) is one of the metrics that measure the reputation of journals. The impact factor is computed based on the average number of citations of research articles published in a journal. If articles are cited often, the impact factor of a source journal will be higher. We use this metric to measure the reputation of an article. We scored a journal’s reputation from 1 to 10. The score of an article published in a journal that does not have an impact factor is set to 1. The reputation score is defined as

$$Q_{d}=1+\frac{IF_{d}}{max(IF)}\times 9$$

where *IF*_*d*_ is the impact factor of the journal where article *d* is published and *max*(*IF*) is the maximum IF value in the BEST corpus.

#### 4) Recency score (*R*_*d*_)

In the search domain, page recency is one of the most important factors for better user experience [22]. Therefore, BEST gives more weight to new articles. The recency score is computed based on the publication date of a paper. The older the publication date is, the lower the recency score. If an article’s publication date is in the same month as the date of a user’s query, the recency score is 1. An article published two years prior to the query will have a recency score of 0.5. The recency score reduces to half every two years. The recency score of an article older than 8 years is set to 0.0625 (lower bounded). The recency score is defined as

$$R_{d}=1/2^{\frac{min(M-m_{d},96)}{24}}$$

where *M* is the current time in months and *m*_*d*_ is the month when document *d* was published.

Above four scoring components are combined to give the score to each article. The term match score represents the relevance between query and article, and the entity number score reflects the dispersion of importance to the entities, which co-occurred in same article. Thanks to the reputation score and the recency score, the better reputed and newer information is preferred. All the parameters in our scoring model including the lower bounds were selected through the extensive qualitative analysis of query results.

#### PubTator

PubTator is a web-based text-mining application that assists in the manual annotation of biomedical entities in PubMed abstracts.[15] It employs a variety of text-mining tools for named-entity recognition of genes, chemicals, diseases, species, and mutations. For the evaluation of our entity extractor, we used PubTator as the current state-of-the-art baseline. We used PubTator’s API for extracting biomedical entities from the abstracts.

#### BRONCO

We used the recently developed Biomedical entity Relation ONcology COrpus (BRONCO)[14] for comparing the entity extraction accuracy of BEST with that of PubTator. BRONCO is manually curated and contains more than 400 variants and their relations with genes, diseases, drugs, and cell lines in the context of cancer and anti-tumor drug screening research. The variants and relations were manually extracted from 108 full-text articles. In this study, we created BRONCO-A which is a corpus of all the abstracts in BRONCO, as PubTator performs biomedical entity extraction only on abstracts. As BRONCO-A is manually curated, we have the “true answers” to evaluating the precision and recall of the entity extraction methods. BRONCO is freely available at http://infos.korea.ac.kr/bronco.

#### Precision, Recall, and F1-score

To evaluate the biomedical entity extraction accuracy of BEST and PubTator, we used precision, recall, and F1-score as the evaluation metrics. Precision and recall are defined as

$$Precision=\frac{TP}{TP+FP},\;Recall=\frac{TP}{TP+FN}$$

where TP, FP, and FN are true positives, false positives, and false negatives, respectively. F1-score is the harmonic mean of precision and recall and is defined as

$$F1=2\times \frac{Precision\times Recall}{Precision+Recall}.$$

## Supporting Information

S1 File Supporting information file. (DOCX)

## References

[1] Cohen KB, Hunter LE (2013) Chapter 16: Text Mining for Translational Bioinformatics. Plos Computational Biology 9. 10.1371/journal.pcbi.1003044 PMC3635962 23633944

[2] Tsuruoka Y, Tsujii J, Ananiadou S (2008) FACTA: a text search engine for finding associated biomedical concepts. Bioinformatics 24: 2559–2560. 10.1093/bioinformatics/btn469 18772154 PMC2572701

[3] Tsuruoka Y, Miwa M, Hamamoto K, Tsujii J, Ananiadou S (2011) Discovering and visualizing indirect associations between biomedical concepts. Bioinformatics 27: I111–I119. 10.1093/bioinformatics/btr214 21685059 PMC3117364

[4] Kim J, So S, Lee HJ, Park JC, Kim JJ, Lee H (2013) DigSee: Disease gene search engine with evidence sentences (version cancer). Nucleic Acids Res 41: W510–517. 10.1093/nar/gkt531 23761452 PMC3692119

[5] Lee HJ, Dang TC, Lee H, Park JC (2014) OncoSearch: cancer gene search engine with literature evidence. Nucleic Acids Res 42: W416–421. 10.1093/nar/gku368 24813447 PMC4086113

[6] Plake C, Schiemann T, Pankalla M, Hakenberg J, Leser U (2006) ALIBABA: PubMed as a graph. Bioinformatics 22: 2444–2445. 10.1093/bioinformatics/btl408 16870931

[7] Cheng D, Knox C, Young N, Stothard P, Damaraju S, Wishart DS (2008) PolySearch: a web-based text mining system for extracting relationships between human diseases, genes, mutations, drugs and metabolites. Nucleic Acids Research 36: W399–W405. 10.1093/nar/gkn296 18487273 PMC2447794

[8] Liu YF, Liang YJ, Wishart D (2015) PolySearch2: a significantly improved text-mining system for discovering associations between human diseases, genes, drugs, metabolites, toxins and more. Nucleic Acids Research 43: W535–W542. 10.1093/nar/gkv383 25925572 PMC4489268

[9] Choi J, Kim D, Kim S, Lee S, Lee K, Kang J (2012) BOSS: context-enhanced search for biomedical objects. Bmc Medical Informatics and Decision Making 12. 10.1186/1472-6947-12-S1-S7 PMC3339395 22595092

[10] O'Hare T, Eide CA, Deininger MW (2007) Bcr-Abl kinase domain mutations, drug resistance, and the road to a cure for chronic myeloid leukemia. Blood 110: 2242–2249. 10.1182/blood-2007-03-066936 17496200

[11] Soverini S, Hochhaus A, Nicolini FE, Gruber F, Lange T, Saglio G, et al (2011) Bcr-Abl kinase domain mutation analysis in chronic myeloid leukemia patients treated with tyrosine kinase inihibitors: recommendations from an expert panel on behalf of European LeukemiaNet. Blood: blood-2010-2012-326405. 10.1182/blood-2010-12-326405 21562040

[12] Elias MH, Baba AA, Azlan H, Rosline H, Sim GA, Padmini M, et al (2014) BCR-ABL kinase domain mutations, including 2 novel mutations in imatinib resistant Malaysian chronic myeloid leukemia patients—Frequency and clinical outcome. Leukemia research 38: 454–459. 10.1016/j.leukres.2013.12.025 24456693

[13] Jabbour E, Hochhaus A, Cortes J, La Rosee P, Kantarjian HM (2010) Choosing the best treatment strategy for chronic myeloid leukemia patients resistant to imatinib: weighing the efficacy and safety of individual drugs with BCR-ABL mutations and patient history. Leukemia 24: 6–12. 10.1038/leu.2009.193 19798095

[14] Lee K, Lee S, Kim S, Park S, Kim S, Choi K, et al (2016) BRONCO: Biomedical entity Relation ONcology Corpus for Extracting Gene-Variant-Disease-Drug Relations. Database. 10.1093/database/baw043 PMC4830473 27074804

[15] Wei CH, Kao HY, Lu Z (2013) PubTator: a web-based text mining tool for assisting biocuration. Nucleic Acids Res 41: W518–522. 10.1093/nar/gkt441 23703206 PMC3692066

[16] NCI Drugs Approved for Leukemia. National Cancer Institute.

[17] Hehlmann R, Heimpel H, Hasford J, Kolb HJ, Pralle H, Hossfeld DK, et al (1993) Randomized comparison of busulfan and hydroxyurea in chronic myelogenous leukemia: prolongation of survival by hydroxyurea. The German CML Study Group. Blood 82: 398–407. 8329700

[18] Wei CH, Harris BR, Kao HY, Lu ZY (2013) tmVar: a text mining approach for extracting sequence variants in biomedical literature. Bioinformatics 29: 1433–1439. 10.1093/bioinformatics/btt156 23564842 PMC3661051

[19] Jeon M, Lee S, Lee K, Tan AC, Kang J (2014) BEReX: Biomedical Entity-Relationship eXplorer. Bioinformatics 30: 135–136. 10.1093/bioinformatics/btt598 24149052

[20] Eden E, Navon R, Steinfeld I, Lipson D, Yakhini Z (2009) GOrilla: a tool for discovery and visualization of enriched GO terms in ranked gene lists. Bmc Bioinformatics 10. 10.1186/1471-2105-10-48 PMC2644678 19192299

[21] Plikus MV, Zhang Z, Chuong CM (2006) PubFocus: semantic MEDLINE/PubMed citations analytics through integration of controlled biomedical dictionaries and ranking algorithm. Bmc Bioinformatics 7. 10.1186/1471-2105-7-424 PMC1618408 17014720

[22] Dong A, Chang Y, Zheng Z, Mishne G, Bai J, Zhang R, et al. (2010) Towards recency ranking in web search. The third ACM international conference on Web search and data mining, WSDM '10. New York, NY, USA: ACM.

