# Supplementary material for: BEST: Next-Generation Biomedical Entity Search Tool for Knowledge Discovery from Biomedical Literature
Source: PLoS One. 2016 Oct 19;11(10):e0164680. doi: 10.1371/journal.pone.0164680 (PMC5070740; doi:10.1371/journal.pone.0164680)
Supplement: S1 File — (DOCX) [file pone.0164680.s001.docx]

**BEST: Next-Generation Biomedical Entity Search Tool for Knowledge Discovery from Biomedical Literature**

Sunwon Lee^1, +^, Donghyeon Kim^1, +^, Kyubum Lee^1^, Jaehoon Choi^1^, Seongsoon Kim^1^,

Minji Jeon^1^, Sangrak Lim^1^, Donghee Choi^1^, Sunkyu Kim^1^, Aik-Choon Tan^2^, and Jaewoo Kang^1,*^

^1^Department of Computer Science and Engineering, Korea University, Seoul, Korea

^2^Translational Bioinformatics and Cancer Systems Biology Laboratory, Division of Medical Oncology, University of Colorado Anschutz Medical Campus, Aurora, CO, USA

^*^Corresponding author

E-mail: kangj@korea.ac.kr

^+^These authors contributed equally to this work

**Table A. Top 10 drugs returned for query “lung cancer”**

| **Rank** | **BEST** | **FACTA+** | **PolySearch2** | **FDA approved for lung cancer** |
| --- | --- | --- | --- | --- |
| 1  2  3  4  5  6  7  8  9  10 | **cisplatin**  **erlotinib**  **gefitinib**  **paclitaxel**  **docetaxel**  **gemcitabine**  mta  **crizotinib**  **bevacizumab**  **doxorubicin** | **etoposide**  cyclophosphamide  glutathione  progesterone  gel  **gefitinib**  vindesine  vindesine  freeze  **taxol (paclitaxel)** | chlordiazepoxide  omeprazole  levothyroxine  **erlotinib**  vitamin a  **cisplatin**  nicotine  **gemcitabine**  irinotecan  **etoposide** | Afatinib Dimaleate  Alectinib  Bevacizumab  Carboplatin  CARBOPLATIN-TAXOL  Ceritinib  Crizotinib  Docetaxel  Doxorubicin Hydrochloride  Erlotinib Hydrochloride  Etoposide  Etoposide Phosphate  Everolimus  Gefitinib  Gemcitabine Hydrochloride  GEMCITABINE-CISPLATIN  Mechlorethamine Hydrochloride  Methotrexate  Necitumumab  Nivolumab  Osimertinib  Paclitaxel  Paclitaxel Albumin-stabilized Nanoparticle Formulation  Pembrolizumab  Pemetrexed Disodium  Ramucirumab  Topotecan Hydrochloride  Vinorelbine Tartrate |
| **Accuracy** | **9** | 3 (taxol is a brand name of paclitaxel) | 4 | cisplatin is approved as a combinatorial treatment with gemcitabine for lung cancer |
| **Response Time** | 0.116s | 0.09s | 30s |  |

(Answer set comes from following site: http://www.cancer.gov/about-cancer/treatment/drugs/lung)

**Table B. Top 10 drugs returned for query “melanoma”**

| **Rank** | **BEST** | **FACTA+** | **PolySearch2** | **FDA approved for melanoma** |
| --- | --- | --- | --- | --- |
| 1  2  3  4  5  6  7  8  9  10 | **vemurafenib**  **ipilimumab**  **dacarbazine**  **dabrafenib**  cisplatin  **trametinib**  temozolomide  doxorubicin  interferon α  paclitaxel | IFN-gamma  **IL-2 (aldesleukin)**  gel  fluorescein  fluorescein  progesterone  cyclophosphamide  CSF  glutathione  tretinoin | omeprazole  diazepam  **dacarbazine**  chlordiazepoxide  nonoxynol-9  levodopa  sulfacetamide  metronidazole  amitriptyline  levothyroxine | Aldesleukin  Cobimetinib  Dabrafenib  Dacarbazine  Ipilimumab  Nivolumab  Peginterferon Alfa-2b  Pembrolizumab  Recombinant Interferon Alfa-2b  Talimogene Laherparepvec  Trametinib  Vemurafenib |
| **Accuracy** | **5** | 1 (IL-2 is a brand name of aldesleukin) | 1 |  |
| **Response Time** | 0.058s | 0.07s | 28s |  |

(Answer set comes from following site: http://www.cancer.gov/about-cancer/treatment/drugs/melanoma)

**Table C. Top 10 drugs for query “tyrosine kinase inhibitor”**

| **Rank** | **BEST** | **FACTA+** | **PolySearch2** | **Tyrosine kinase inhibitors** |
| --- | --- | --- | --- | --- |
| **1**  **2**  **3**  **4**  **5**  **6**  **7**  **8**  **9**  **10** | **imatinib**  **gefitinib**  **erlotinib**  **sunitinib**  **dasatinib**  **sorafenib**  **nilotinib**  **crizotinib**  **lapatinib**  rituximab | progesterone  **imatinib**  **gleevec**  nitric oxide  **gefitinib**  gel  angiotensin II  forskolin  arachidonic acid  IL-2 | **gefitinib**  **imatinib**  genistein  **sunitinib**  l-tyrosine  **erlotinib**  **nilotinib**  **lapatinib**  **dasatinib**  **pazopanib** | axitinib  bortezomib  bosutinib  carfilzomib  crizotinib  dabrafenib  dasatinib  erlotinib  gefitinib  ibrutinib  imatinib  lapatinib  nilotinib  palbociclib  pazopanib  pegaptanib  ponatinib  regorafenib  ruxolitinib  sorefenib  sunitinib  tofacitinib  trametinib  vandetanib  vemurafenib  vismodegib |
| **Accuracy** | **9** | 2 (gleevec is a brand name of imatinib) | 8 |  |
| **Response Time** | 0.067s | 0.03s | 45s |  |

(Answer set comes from following site: http://livertox.nih.gov/TyrosineKinaseReceptorInhibitors.htm)

**Table D. Functionality comparison of different biomedical entity search systems.**

| Functionality | **BEST**  http://best.korea.ac.kr | **FACTA+**  http://www.nactem.ac.uk/facta/ | **DigSee**  http://gcancer.org/geneSearch/ | **OncoSearch**  http://oncosearch.biopathway.org/ | **AliBaba** | **PolySearch2**  http://polysearch.cs.ualberta.ca/ |
| --- | --- | --- | --- | --- | --- | --- |
| Ad-hoc free-text query | Yes | Yes | No | No | Yes | Yes |
| Information preprocessing | Yes | Yes | Yes | Yes | No | Yes |
| Daily update | Yes | No | No | No | No | Yes |
| Linked entities in result page | Yes | No | No | Yes | Yes | No |
| Real-time response  (< 100ms) | Yes | Yes | Yes | Yes | No | No |

**Table E. BEST index statistics. (July, 07, 2016)**

| **Name** | **Statistics** |
| --- | --- |
| Size of index | 34.22 GB |
| Number of abstracts | 11,882,670 |
| Number of entities | 282,936 |
| Number of abstracts containing genes | 6,631,752 |
| Number of abstracts containing targets | 4,000,775 |
| Number of abstracts containing transcription factors | 805,406 |
| Number of abstracts containing miRNAs | 20,360 |
| Number of abstracts containing chemical compounds | 6,722,908 |
| Number of abstracts containing drugs | 3,769,971 |
| Number of abstracts containing toxins | 3,618,492 |
| Number of abstracts containing diseases | 4,272,957 |
| Number of abstracts containing pathways | 25,932 |
| Number of abstracts containing mutations | 252,629 |
| Number of abstracts containing cell lines | 2,917,171 |

**Fig A.** **Entity information page of “chronic myeloid leukemia.”** Every entity in BEST has a designated page containing more detailed information about the entity. An entity page can be reached by clicking the entity name in the main result page. The entity page presents additional information including (a) phrases that are most frequently co-occur with the current entity and the query terms and (b) the related keyword list consisting of most frequent “related substances” extracted from the matching articles. Through this page, users also can retrieve all abstracts in which the query terms and the entity co-occur.

**
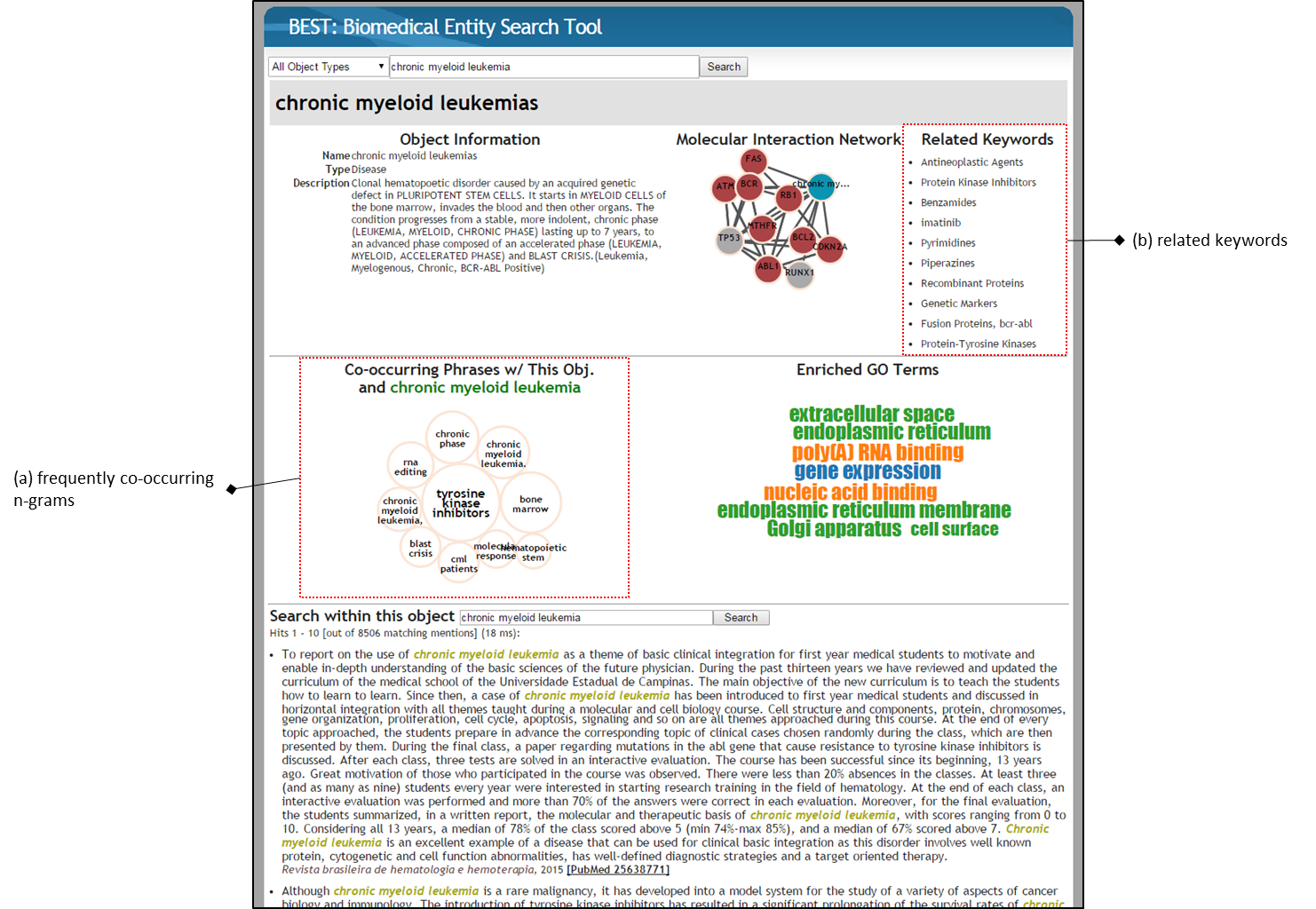
**
